# The Importance of the Gut–Muscle Axis: From Mechanistic Insights in Cell Culture and Rodent Models to Descriptive and Associative Evidence in Livestock

**DOI:** 10.3390/ani16162594

**Published:** 2026-08-19

**Authors:** Robert Ringseis, Klaus Eder, Denise K. Gessner

**Affiliations:** 1 Institute of Animal Nutrition and Nutrition Physiology, Justus Liebig University Giessen, Heinrich-Buff-Ring 26–32, 35392 Giessen, Germany; klaus.eder@ernaehrung.uni-giessen.de (K.E.); denise.gessner@ernaehrung.uni-giessen.de (D.K.G.); 2 Center for Sustainable Food Systems, Justus Liebig University Giessen, Senkenbergstraße 3, 35390 Giessen, Germany

**Keywords:** gut microbiota, meat quality, microbial metabolites, monogastrics, ruminants

## Abstract

The gut microbiota is a metabolically active ecosystem that influences host physiology through bioactive metabolites and interactions with host signaling pathways. Recent research has established a bidirectional gut–muscle axis in which microbial metabolites and muscle-derived factors (myokines) regulate muscle protein synthesis, degradation, regeneration, fiber-type specification, and overall muscle performance. Studies using germ-free, antibiotic-treated, probiotic-supplemented, and fecal microbiota transplantation models demonstrate that the gut microbiota is a critical determinant of skeletal muscle mass and function. Key mediators include short-chain fatty acids, bile acids, aromatic amino acid metabolites, microbial-associated molecular patterns, and methylamine metabolites. This review summarizes current mechanistic knowledge of gut–muscle communication and its relevance to livestock production. In monogastric livestock, particularly pigs and poultry, microbiota transplantation experiments and targeted probiotic interventions provide causal evidence that gut microbial communities influence muscle growth, muscle fiber composition, intramuscular fat deposition, carcass traits, and meat quality, including tenderness, marbling, water-holding capacity, and flavor. Several studies have also identified specific microbial taxa and metabolites capable of transferring desirable production phenotypes. In contrast, evidence in ruminants remains largely associative and originates mainly from multi-omics and dietary intervention studies. Future research should validate causal mechanisms, identify robust microbial biomarkers, and develop species-specific microbiome-based strategies for precision livestock production.

## Simple Summary

The gut microbiota, the community of microorganisms inhabiting the gastrointestinal tract, is increasingly recognized as a key regulator of skeletal muscle growth, metabolism, and function through a bidirectional gut–muscle axis. Microbial metabolites, including short-chain fatty acids and bile acids, influence muscle protein synthesis, fiber-type composition, and energy metabolism, while muscle-derived signaling molecules can affect intestinal function and microbial composition. Experimental studies show that disruption of the gut microbiota impairs muscle development and performance, whereas microbiota transplantation and probiotic supplementation can improve muscle mass and function. In livestock, however, the strength of evidence differs among species. In monogastric animals such as pigs and poultry, fecal or cecal microbiota transplantation and targeted probiotic interventions provide causal evidence that gut microbial communities regulate intramuscular fat deposition, muscle fiber characteristics, carcass traits, and meat quality attributes including marbling, tenderness, water-holding capacity, and flavor. In contrast, evidence in ruminants is derived largely from microbiome–metabolome association studies and dietary interventions, which reveal links between microbial metabolites, muscle gene expression, and meat quality but have not yet established causality. This review summarizes current knowledge of the gut–muscle axis, highlights key metabolites and signaling pathways involved in muscle regulation, and discusses opportunities for microbiome-based strategies to improve livestock performance and meat quality.

## 1. Introduction

The gastrointestinal tract of animals harbors a complex and dynamic ecosystem composed of bacteria, archaea, fungi, and viruses, collectively referred to as the gut microbiota. In recent years, numerous studies in both experimental animals and humans have deliberately manipulated the gut microbiota through fecal microbiota transplantation (FMT) or the administration of probiotics, prebiotics, or antibiotics. These studies have revealed the profound impact of the gut microbiota on host metabolism and overall health [1]. A striking example is the identification of an obesogenic gut microbiota capable of inducing obesity when transferred from an obese donor to a germ-free, lean recipient animal [2,3]. Several microbiota-derived metabolites acting as communication signals, along with diverse host receptors that sense these molecules, have been identified as key mechanisms underlying the crosstalk between the gut microbiota and the host [4]. Initial investigations into microbiota–host crosstalk predominantly focused on the gut–liver axis, reflecting the liver’s role as the primary site of exposure to gut-derived microbial constituents [5]. However, it has since been conclusively demonstrated that the gut microbiota exerts widespread systemic effects, influencing the functional integrity and physiological homeostasis of essentially all organs and tissues within the host.

In the most recent phase of research on microbiota–host interactions, increasing attention has been directed toward the interplay between the gut and skeletal muscle, known as the gut–muscle axis. Remarkably, the identification of muscle-derived secretory factors, termed myokines, which can influence the gut by modulating microbial composition and intestinal function [6], has revealed that communication between the gut and skeletal muscle is bidirectional and that both organs mutually affect each other. Convincing evidence for the existence of a gut–muscle axis has been established in studies using mice in which the gut microbiota was depleted through either germ-free husbandry or broad-spectrum antibiotic treatment. Such depletion impaired muscle function, as evidenced by muscle atrophy, increased fatigability, and reduced endurance performance [7,8]. Mechanistic studies in germ-free mice revealed inhibition of protein anabolic pathways, stimulation of proteasome-dependent muscle protein degradation, and downregulation of genes involved in myogenic differentiation in skeletal muscle [7]. These findings indicate that the gut microbiota is a critical regulator of muscle physiology by influencing protein synthesis, protein degradation, and muscle regeneration processes. In addition, several studies demonstrated that the administration of probiotics increases muscle mass and muscle strength and improves endurance performance in mice [9,10,11,12]. Evidence in support of a gut–muscle axis has also been provided from FMT experiments, in which muscle strength was shown to be increased in germ-free mice receiving fecal samples from high-functioning older adults when compared to mice colonized with fecal samples from low-functioning older adults [13]. FMT from young to aged mice has been reported to increase muscle mass and improve muscle strength, thereby preventing aging-induced sarcopenia [14]. This effect is likely attributable to the reversal of aging-induced microbial dysbiosis, which is characterized by the loss of symbiotic bacterial taxa and the expansion of pro-inflammatory pathobionts.

Apart from muscle mass and strength, muscle fiber composition, which is closely related to meat quality, was also demonstrated to be regulated by the gut–muscle axis [15,16,17,18]. Meat quality is commonly assessed based on marbling, tenderness, water-holding capacity, pH, color, and flavor. Marbling, which reflects intramuscular fat content, enhances juiciness, tenderness, and flavor, thereby increasing meat value. Tenderness is a key determinant of consumer acceptance, while water-holding capacity influences both processing yield and juiciness. Meat pH affects color, tenderness, shelf life, and water-holding capacity. Color strongly influences purchasing decisions, whereas flavor is a major contributor to eating quality. Together, these traits are evaluated using visual, instrumental, and sensory methods and largely determine the economic value of meat products [19,20]. Collectively, this indicates that the gut microbiota–muscle crosstalk is also of great importance for meat-producing livestock and underscores the potential of targeting muscle function and improving meat quality by modulating the gut microbiota composition.

Considering this, the present review aims to (1) provide emerging knowledge from in vitro and rodent models on the crucial role of the gut microbiota in skeletal muscle function and the mechanisms underlying gut–muscle crosstalk. (2) This review summarizes current evidence on the regulation of muscle function and meat quality traits through the gut–muscle axis in monogastric and ruminant livestock.

## 2. Mechanistic Insights into the Gut–Muscle Axis from In Vitro and Rodent Models

**Figure 1:**
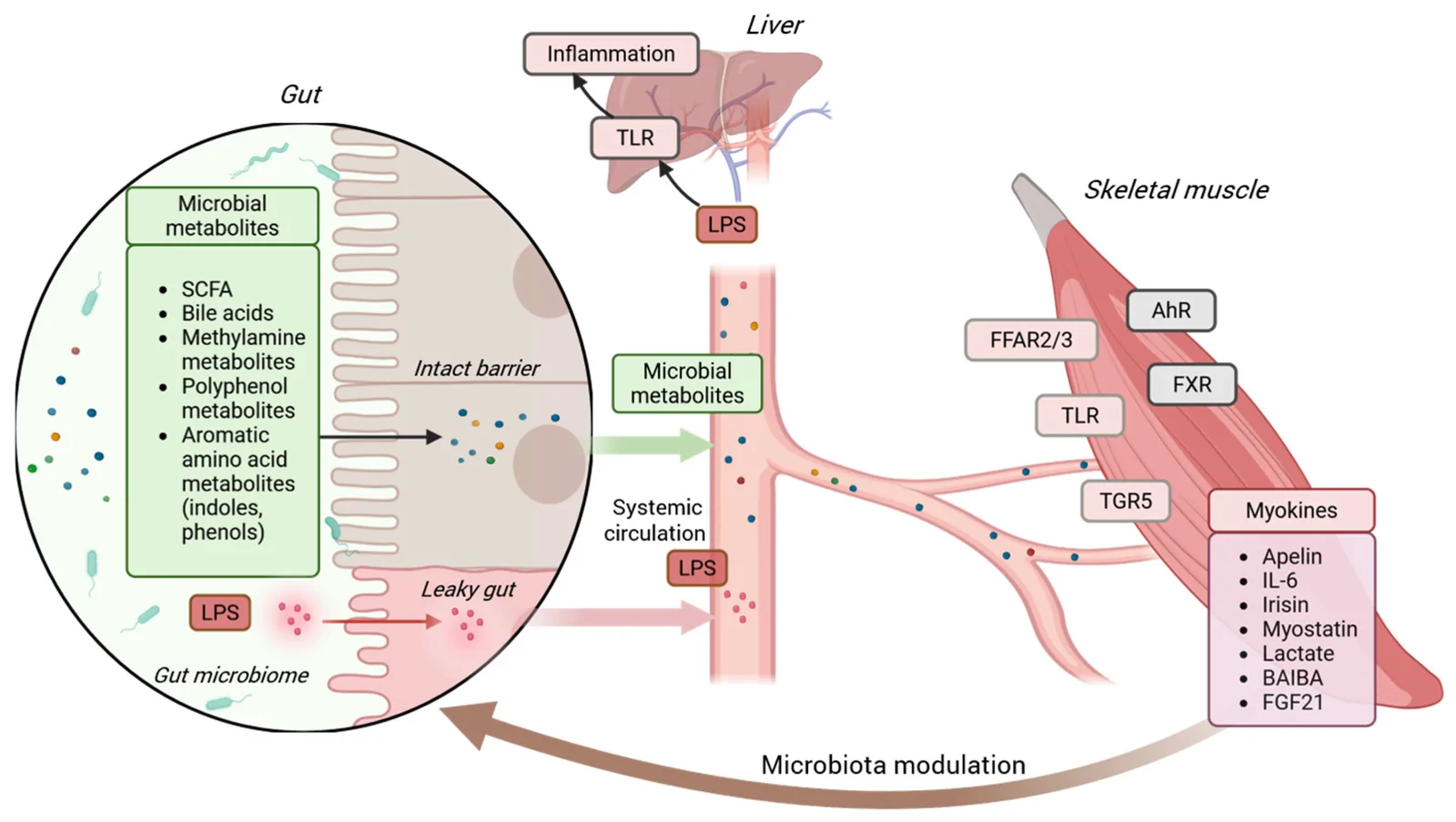
The bidirectional gut microbiota–skeletal muscle crosstalk. The crosstalk between the gut microbiota and skeletal muscle is mediated by a variety of gut-derived signaling molecules that can enter the systemic circulation, as well as different host receptors, which sense these signals thereby stimulating signaling and metabolic pathways in skeletal muscle. While some of these molecules are directly produced by gut microbes, such as short-chain fatty acids (SCFA), others, like bile acids, methylamine metabolites and polyphenol-derived metabolites, are formed from cooperative metabolic interactions between the gut microbiota and the host. Microbial-associated molecular patterns including lipopolysaccharide (LPS) translocate into the portal vein and subsequently into the systemic circulation because of impaired gut barrier function (“leaky gut”). In the liver, LPS is recognized by specific pattern-recognition receptors, such as Toll-like receptors (TLR), thereby triggering inflammatory signaling pathways and causing secretion of proinflammatory cytokines into the circulation. In skeletal muscle, the proinflammatory cytokines promote muscle atrophy by inducing the expression of muscle-specific E3 ubiquitin ligases, which are key regulators of ubiquitin–proteasome-mediated proteolysis. In addition, host-derived factors, such as myokines, also participate in the gut microbiota–host communication, highlighting the bidirectional nature of the gut-muscle axis. Through the effects of myokines, such as apelin, interleukin-6 (IL-6), irisin, myostatin, lactate, β-aminoisobutyric acid (BAIBA) and fibroblast growth factor 21 (FGF21), on the gut, the positive influence of regular exercise on the composition, the diversity and the functionality of the gut microbiota and the gut mucosal integrity compared with the absence of muscular load is rendered comprehensible. Created in BioRender. Eder, K. (2026) https://BioRender.com/0t5782e.

The crosstalk between the gut microbiota and skeletal muscle is mediated by a variety of gut-derived signaling molecules that can cross the intestinal barrier and enter the systemic circulation. As described below, some of these molecules are directly produced by gut microbes, such as short-chain fatty acids (SCFA), or represent bacterial components, such as lipopolysaccharides (LPS), lipoteichoic acid, peptidoglycan, flagellin and bacterial desoxy- and ribonucleic acids, together referred to as microbial-associated molecular patterns (MAMP). Others, including bile acids, methylamine metabolites, and polyphenol-derived metabolites, result from cooperative metabolic interactions between the gut microbiota and the host. In addition, host-derived factors, such as myokines, also participate in this communication, highlighting the bidirectional nature of the gut–muscle axis, which is schematically summarized in Figure 1.

### 2.1. Gut Microbiota-Derived Signals

*Bile acids.* Beyond their detergent role in intestinal lipid absorption, bile acids are important signaling molecules with local and systemic effects [21]. Primary bile acids, including cholic acid (CA), chenodeoxycholic acid (CDCA), and α-/β-muricholic acid (MCA), are synthesized in the liver, secreted into the intestine as glycine or taurine conjugates, and largely recycled through enterohepatic circulation [22]. Less than 5% escape ileal reabsorption and are converted by gut microbes into secondary bile acids such as deoxycholic acid (DCA), lithocholic acid (LCA), murideoxycholic acid (MDCA), hyodeoxycholic acid (HDCA), and ursodeoxycholic acid (UDCA). A small fraction enters the systemic circulation and mediates signaling in peripheral tissues. The two best-characterized bile acid receptors involved in skeletal muscle regulation are the farnesoid X receptor (FXR) [23] and Takeda G-protein-coupled receptor 5 (TGR5) [24]. TGR5 activation enhances energy expenditure through deiodinase 2-mediated T4-to-T3 conversion in skeletal muscle [25]. Correspondingly, TGR5-deficient mice exhibit reduced muscle mass and grip strength together with increased expression of atrophy-related E3 ubiquitin ligases, whereas muscle-specific TGR5 overexpression increases muscle mass and strength [26]. Exercise-induced endoplasmic reticulum stress also upregulates TGR5, suggesting a role in muscular adaptation to exercise [26]. In vitro, the potent TGR5 ligands taurolithocholic acid (TLCA) and LCA stimulate myogenic differentiation, activate Akt/mTOR signaling, suppress FOXO3-mediated atrophy pathways, and increase expression of myogenic markers [26,27]. In contrast, CA and DCA induce atrophy-like changes in C2C12 myotubes and isolated muscle fibers via TGR5-dependent mechanisms [28]. These discrepant findings may be explained by differences in TGR5 agonistic potency, as LCA and TLCA are stronger activators than DCA and CA [24], as well as by variation in cellular TGR5 expression. FXR represents another key mediator of gut microbiota–host communication. Because different bile acids act as FXR agonists or antagonists, for example, UDCA and tauro-β-MCA (TβMCA) [29], microbiota-driven changes in bile acid composition can markedly influence FXR signaling. Intestinal FXR regulates metabolism partly through fibroblast growth factor (FGF)15/19 secretion, which influences energy balance and protein synthesis [30]. Antibiotic-induced microbiota depletion impairs bile acid metabolism, increases TβMCA levels, suppresses FXR-FGF15/19 signaling, and causes muscle atrophy through reduced extracellular signal-regulated protein kinase 1/2 (ERK1/2)-mediated protein synthesis [31]. Importantly, FGF19 treatment partially reverses muscle loss, highlighting the importance of the FXR-FGF15/19 axis. Consistently, activation of ileal FXR signaling with the gut-specific agonist fexaramine improves muscle mass and function in aged mice by restoring age-related declines in FGF15/19 signaling [31].

*SCFA.* SCFA are the major microbial metabolites generated during fermentation of indigestible carbohydrates. Acetate, propionate, and butyrate account for over 95% of total SCFA, while valerate, isovalerate, formate, and caproate are present in smaller amounts. SCFA serve as energy substrates for colonocytes, support epithelial health, and enter both portal and systemic circulation, where they influence host metabolism [32,33,34]. The identification of SCFA receptors—free fatty acid receptor (FFAR2)/G-protein coupled receptor (GPR)43, FFAR3/GPR41, and GPR109A—in skeletal muscle and other tissues suggests direct gut–muscle communication [35,36]. Dietary butyrate enhances mitochondrial biogenesis, fatty acid oxidation, and energy expenditure in skeletal muscle, contributing to obesity prevention and potentially promoting oxidative type I fibers [37]. SCFA also indirectly support muscle health by maintaining gut barrier integrity. Butyrate enhances expression of tight-junction proteins and mucins, whereas SCFA depletion compromises barrier function, facilitating translocation of MAMP and promoting muscle proteolysis through ubiquitin–proteasome pathways [38,39,40]. Cachexia is characterized by loss of SCFA-producing bacteria, reduced butyrate and acetate levels, and enrichment of pro-inflammatory pathobionts [41,42,43]. Similarly, cachectic patients exhibit lower fecal SCFA concentrations than controls [44]. Interventions targeting microbiota support a causal role for SCFA. Administration of *Lactobacillus (L.) reuteri* reduces inflammatory and atrophy markers while increasing muscle mass in cachectic mice [45,46]. Likewise, a postbiotic mixture of acetate, butyrate, and propionate increased muscle mass and reduced expression of E3 ubiquitin ligases in germ-free mice [7].

*MAMP.* The MAMP including LPS can enter the circulation when intestinal barrier integrity is impaired (“leaky gut”). In the liver, these molecules activate pattern-recognition receptors such as TLRs, triggering nuclear factor-kappa B (NF-κB) signaling and inducing the production of pro-inflammatory cytokines and chemokines [47]. Both circulating MAMP and liver-derived inflammatory mediators contribute to systemic inflammation affecting the brain and skeletal muscle. Hypothalamic inflammation disrupts appetite regulation and energy balance [4], while skeletal muscle inflammation induces expression of the E3 ubiquitin ligases, such as muscle RING finger protein-1 (MuRF1) and atrogin-1, promoting proteasomal protein degradation [39,40]. Activation of the hypothalamic–pituitary–adrenal axis further increases cortisol secretion and muscle proteolysis [48]. Thus, preservation of gut barrier integrity is crucial for maintaining muscle mass.

*Microbial aromatic amino acid metabolites.* Proteins reaching the colon are fermented by gut microbes into a variety of metabolites derived from phenylalanine, tyrosine, and tryptophan [49,50]. Several tryptophan-derived metabolites, including indole and indole-3-propionic acid (IPA), regulate epithelial integrity, barrier function, and mucosal immune responses through receptors such as the aryl hydrocarbon receptor and pregnane X receptor [51,52,53,54,55]. These metabolites can enter the circulation and influence distant organs; for example, indole has been shown to reduce hepatic inflammation in obese mice [56]. Emerging evidence also links microbial aromatic amino acid metabolites to skeletal muscle regulation. IPA stimulates the expression of myogenic regulatory factors in C2C12 myotubes and mediates muscle-promoting effects of *Clostridium (C.) sporogenes* [57]. Consistent with this, both *C. sporogenes* and IPA counteracted antibiotic-induced muscle atrophy in mice by restoring the expression of myogenic and atrophy-related genes [58]. Another microbial metabolite, 3-phenylpropionic acid (3-PPA), increased muscle weight and myotube diameter by inhibiting FOXO3 activity and thereby suppressing protein degradation pathways [59]. Reduced expression of mitochondrial enzyme genes further suggests that 3-PPA may promote hypertrophic type II fibers at the expense of oxidative type I fibers [59].

*Microbial polyphenol metabolites.* Polyphenols comprise more than 8000 plant-derived compounds, most of which escape absorption in the small intestine and undergo microbial transformation in the colon [60,61,62]. The resulting metabolites exert several beneficial effects on skeletal muscle. Isovanillic acid 3-O-sulfate and dihydroferulic acid 4-O-sulfate enhance glucose uptake in human myoblasts through insulin-like actions [63]. Urolithin A suppresses NF-κB activation and proteasomal protein degradation [64], whereas urolithin B promotes mTOR-dependent protein synthesis while inhibiting protein degradation [65]. Furthermore, hippurate enhances glucose oxidation in primary human skeletal muscle cells [66].

*Microbial methylamine metabolites.* Various gut bacteria convert choline, L-carnitine, and betaine into trimethylamine (TMA), which is subsequently oxidized in the liver by flavin-containing monooxygenase 3 to trimethylamine-N-oxide (TMAO) [67,68,69,70]. Although TMAO is widely recognized as a cardiovascular risk factor [71], its effects on skeletal muscle remain controversial. TMAO aggravated high-fat diet-induced reductions in muscle mass, strength, and function while inhibiting Akt/mTOR signaling, suggesting a role in sarcopenic obesity [72]. Elevated plasma TMAO has also been associated with woody breast myopathy in broilers [73]. In contrast, other studies reported improved exercise performance and protection against oxidative stress through activation of Nrf2-dependent antioxidant pathways and enhanced glutathione-related defenses [74,75,76]. These apparently conflicting findings may reflect substantial differences in experimental design, including TMAO dosage (1% (*v*/*v*) via drinking water vs. 800 mg/kg body weight), treatment duration (12 d vs. 16 wk), animal age (young vs. aged) and physiological status, dietary background (standard diet vs. high-fat diet (HFD) feeding), species, and investigated endpoints. For example, the detrimental effects reported by Mo et al. [72] were observed in a model of HFD-induced metabolic dysfunction, whereas the studies by Zou et al. [74,75,76] focused on exercise performance and antioxidant responses in otherwise healthy animals. Likewise, the association with woody breast myopathy was based on plasma metabolite concentrations and does not necessarily indicate direct effects within skeletal muscle tissue [73]. Consequently, current evidence does not conclusively support either beneficial or detrimental effects of TMAO on skeletal muscle, suggesting that its actions may depend on physiological and metabolic context. Further studies using standardized experimental approaches are needed to clarify the role of TMAO in muscle physiology. Beyond TMAO, microbiota-derived betaine may influence muscle fiber composition. Yan et al. [16] showed that increased abundance of *Akkermansia muciniphila* elevated betaine levels, promoting a shift from type II to oxidative type I muscle fibers through enhanced N6-methyladenosine RNA methylation and increased expression of myosin heavy chain 7 (MYH7). This effect likely depends on betaine’s role as a methyl donor in S-adenosylmethionine synthesis [77].

### 2.2. Host-Derived Signals: Myokines

Skeletal muscle is now recognized as an endocrine organ that releases regulatory factors known as myokines or exerkines when secreted in response to contraction [78]. These molecules are thought to mediate many health-promoting effects of exercise, supporting the concept of “exercise as medicine” [79]. Myokines act in autocrine, paracrine, and endocrine manners, influencing skeletal muscle, other organs, and the gut [6]. Their effects on gut function help explain the positive impact of exercise on gut microbiota composition, diversity, functionality, and barrier integrity [80], demonstrating the bidirectional nature of the gut–muscle axis. Myokines include peptides (e.g., apelin, irisin, myostatin, FGF21, decorin, follistatin-like 1, SPARC, and Metrnl), metabolites [e.g., β-aminoisobutyric acid (BAIBA), lactate], cytokines (IL6, IL8, and IL15), and non-coding RNAs. Their effects depend on context as follows: exercise-induced myokines such as Metrnl, SPARC, and IL15 generally promote anti-inflammatory and metabolic benefits, whereas sedentary lifestyle, obesity, and chronic inflammation are associated with increased secretion of catabolic factors such as myostatin [81]. Selected myokines with reported effects on the gut and gut microbiota are discussed below.

*Apelin.* Apelin is a contraction-induced peptide derived from a 77-amino-acid precursor and released in several active forms, including apelin-36 and apelin-13 [82,83]. Beyond its role in ovarian physiology [83], apelin is an important regulator of skeletal muscle function [84]. Apelin levels decline with aging, whereas exercise increases circulating apelin. Mice lacking apelin or its receptor develop age-related muscle dysfunction, while restoration of apelin signaling improves muscle function and attenuates sarcopenia through effects on mitochondrial biogenesis, autophagy, inflammation, and muscle stem cell activity [84]. Apelin also affects gut physiology. In rodents, apelin administration inhibits gastric emptying and colonic transit [85] and modulates stress- or corticotropin-releasing factor (CRF)-induced colonic motility [86]. Furthermore, apelin signaling promotes CRF-induced visceral hypersensitivity and colonic hyperpermeability in rats, suggesting a role in irritable bowel syndrome pathophysiology [87]. Prolonged apelin-13 administration additionally impaired gastrointestinal tissue maturation in young rats [88].

*Irisin.* Irisin is generated by proteolytic cleavage of fibronectin type III domain-containing protein 5 (FNDC5), a peroxisome proliferator-activated receptor gamma coactivator 1-α (PGC-1α)-regulated transmembrane protein, and is released during muscular activity. Irisin promotes thermogenesis through browning of white adipose tissue and stimulation of lipolysis [89]. In addition, it enhances mitochondrial biogenesis, increases oxidative capacity of skeletal muscle, and exerts anti-inflammatory effects [90]. In livestock, irisin has been linked to reproduction and energy balance. Plasma irisin concentrations increase during early lactation in dairy cows and are associated with negative energy balance [91], while in vitro studies demonstrated effects on steroidogenesis and ovarian cell metabolism [92,93]. Evidence for gut-related actions derives mainly from a mouse model of ulcerative colitis, where exogenous irisin reduced intestinal and systemic inflammation and altered gut microbiota composition, including changes in *Lactobacillus*, Deferribacteres, and Ruminococcaceae-UCG-014 abundance [94]. These findings suggest that irisin may support intestinal health partly through modulation of the gut microbiota. Notably, FNDC5 may also exert irisin-independent functions, including interactions with FGF21, a metabolic regulator classified as a hepatokine [95] and, under certain conditions, as a myokine [96].

*Myostatin.* Myostatin (growth differentiation factor 8) is a potent negative regulator of muscle growth, regeneration, and maintenance [97]. Consistent with this role, myostatin-knockout mice and cattle carrying myostatin mutations exhibit markedly increased muscle mass (“double muscling”) [98,99]. Recent studies suggest that myostatin also influences the gut–muscle axis. Pigs lacking myostatin display altered gut microbiota composition [100], likely due to intestinal structural changes associated with increased expression of tight-junction genes, thicker muscularis layers, and longer plicae [101]. Importantly, transplantation of microbiota from myostatin-deficient pigs into germ-free mice induced hypertrophy of fast-twitch type II glycolytic muscles, indicating that microbiota-mediated mechanisms contribute to the muscular phenotype [101]. The classification of myostatin as a pro-inflammatory myokine is further supported by its increased expression in chronic inflammatory diseases such as chronic obstructive pulmonary disease [102].

*BAIBA.* The non-proteinogenic amino acid BAIBA originates from valine and thymine metabolism. Its plasma concentration increases in response to aerobic exercise in humans and endurance training in rodents [103,104]. Increased BAIBA secretion by PGC-1α-overexpressing myocytes indicates that exercise-induced BAIBA production is mediated by PGC-1α, a key regulator of mitochondrial metabolism and oxidative muscle fiber formation [105,106]. As an exercise-induced myokine, BAIBA contributes to systemic metabolic adaptations, including browning of white adipose tissue and enhanced hepatic β-oxidation [105]. Although direct effects of BAIBA on the gut microbiota or intestinal function have not yet been demonstrated, its role as a circulating exercise-responsive signal suggests a potential contribution to the bidirectional gut–muscle axis [103,104,105,106]. Supporting its broader metabolic regulatory role, BAIBA stimulates fatty acid oxidation and reduces lipid accumulation in bovine oocytes [107].

*Lactate.* Lactate is considered the most abundant myokine/exerkine because it reaches millimolar concentrations in muscle, blood, and numerous tissues and regulates metabolism in organs including the liver, heart, kidneys, brain, reproductive tissues, and skeletal muscle [108]. The gut mucosa expresses sodium-dependent monocarboxylate transporters capable of transporting lactate across the intestinal epithelium [109], enabling interactions between exercise-derived lactate and gastrointestinal tissues. However, lactate is also generated within the gut following consumption of fermented foods, probiotics, or fiber-rich diets that promote microbial fermentation. Consequently, distinguishing the specific contribution of muscle-derived lactate from gut-derived lactate remains challenging.

Taken together, the available evidence highlights the gut microbiota–muscle axis as a highly integrated and bidirectional communication network. On the one hand, gut-derived signals, including bile acids, SCFA, MAMP, aromatic amino acid metabolites, polyphenol-derived metabolites, and methylamine metabolites, influence skeletal muscle metabolism, protein turnover, fiber-type composition, mitochondrial function, and inflammatory status. On the other hand, muscle-derived signals such as apelin, irisin, myostatin, BAIBA, and lactate can modulate intestinal function, barrier integrity, and gut microbial composition and activity. Thus, skeletal muscle should not be viewed merely as a downstream target of microbial metabolites, nor the gut as a passive source of signals. Rather, both organs continuously interact through multiple endocrine, metabolic, and immunological pathways, forming a dynamic feedback system in which changes in gut microbial ecology can affect muscle physiology, while alterations in muscle activity and metabolic status can reciprocally shape the intestinal environment and microbiota.

## 3. Evidence for Regulation of Muscle Function and Meat Quality Traits via the Gut–Muscle Axis in Livestock

As demonstrated in the preceding chapter, most mechanistic insights into the gut microbiota–muscle crosstalk have been obtained from in vitro systems and rodent models, including wild-type, knockout/transgenic, germ-free, antibiotic-treated, obese, cachectic, and aged mice. Evidence in livestock species is relatively scarce and less mechanistically detailed, though some studies in myostatin-deleted pigs and cattle or “woody breast” broilers suggest that similar gut–muscle interactions may occur. The following sections will summarize the evidence supporting the existence of a functional gut–muscle axis in pigs, poultry and ruminants.

### 3.1. Pigs

The available evidence from studies in pigs as summarized in Table 1 provides compelling support for a functional gut–muscle axis, in which the gut microbiota exerts causal effects on muscle development, fat deposition, and meat quality traits. Unlike many associative studies, FMT experiments offer strong mechanistic insight by demonstrating transferability of phenotypes. For instance, FMT from obese Jinhua pigs to mice induced increased intramuscular triglyceride accumulation and an obese phenotype, whereas FMT from lean Landrace pigs promoted a lean phenotype [110]. Similarly, FMT from Ningxiang pigs to Duroc Landrace Yorkshire (DLY) pigs increased intramuscular fat content and improved meat quality traits, including marbling, meat color, and early postmortem pH [111]. These findings provide direct evidence that gut microbial communities can modulate economically important production traits. Importantly, the causal role of specific microbial taxa has been further substantiated. Yin et al. [111] demonstrated that administration of *L. reuteri* isolated from Ningxiang pigs recapitulated the beneficial effects of FMT, thereby identifying a key effector species. Mechanistically, *L. reuteri* reduced the skeletal muscle expression of the carnitine transporter novel organic cation transporter 2, which is regulated by peroxisome proliferator-activated receptor α [112]. This reduction led to lower muscle carnitine levels and enhanced intramuscular lipid deposition. These findings illustrate how individual bacterial strains can directly influence host metabolic pathways and highlight the translational potential of targeted microbiome interventions.

However, the effects of the gut microbiota on meat quality are not universally beneficial. Chen et al. [113] reported that *Prevotella (P.) copri* abundance was positively associated with carcass fat accumulation but negatively with lean meat percentage in Duroc pigs. Moreover, *P. copri* abundance correlated with increased serum LPS levels, suggesting impaired gut barrier integrity and chronic inflammation. Experimental evidence confirmed that *P. copri* activates TLR4 and mTOR signaling pathways, promoting lipogenesis and fat accumulation while suppressing lipolysis, lipid transport, and muscle growth [113]. These findings underscore that the impact of the microbiota on muscle and carcass traits depends not only on the presence of microbes but also on their functional properties, including their capacity to induce inflammation.

Beyond fat deposition, the gut microbiota also plays a critical role in regulating muscle fiber composition. FMT studies demonstrated that microbiota transfer from obese Rongchang pigs to germ-free mice increased the proportion of slow-twitch type I fibers while reducing fast-twitch type IIb fibers [114], indicating that microbial communities can influence muscle functional properties. In contrast, FMT from myostatin-deletion pigs induced hypertrophy of fast-twitch glycolytic fibers [101]. This effect was linked to increased production of SCFA, particularly valeric acid, which stimulated myoblast differentiation and promoted fast-twitch fiber growth via activation of the Akt/mTOR signaling pathway through the SCFA receptor GPR43 in C2C12 myotubes [101]. At first glance, these findings appear contradictory; however, direct comparison of the two studies is limited by substantial differences in the donor animals and experimental conditions. These include differences in pig breed and genetic background, age and physiological status, dietary regimen, microbiota composition, and potentially the microbial load transferred during FMT. Furthermore, muscle fiber composition was assessed in different experimental contexts and may have varied depending on the muscle examined and the timing of tissue sampling. Notably, the microbiota transferred from obese Rongchang pigs originated from a model characterized by enhanced fat deposition, whereas the microbiota from myostatin-deletion pigs was associated with a pronounced muscle-growth phenotype. Therefore, the divergent fiber-type shifts may reflect distinct microbiota-derived metabolic signals rather than inconsistent effects of the gut microbiota per se. Collectively, these findings suggest that the influence of the gut microbiota on muscle phenotype is highly context-dependent and determined by both host and microbial factors.

In addition to SCFA, other microbial metabolites contribute to the regulation of intramuscular fat deposition. Dietary supplementation with microbiota-derived TMAO increased intramuscular fat content in pigs and induced expression of lipogenic genes such as sterol regulatory element-binding protein 1 and fatty acid synthase in C2C12 myotubes [115]. This suggests that microbial metabolites can directly regulate muscle lipid metabolism through transcriptional control of key anabolic pathways.

Comparative studies across pig breeds further support the role of the gut microbiota in determining meat quality. Songliao Black pigs, characterized by superior meat quality, exhibit distinct gut microbial compositions and metabolite profiles compared with Large White × Landrace (LWL) pigs [116,117]. Integrated transcriptome-microbiome analyses revealed that microbial taxa enriched in Songliao Black pigs are associated with genes promoting muscle protein synthesis and fat deposition, whereas taxa enriched in LWL pigs are linked to glycolytic metabolism and reduced fat accumulation [117]. These findings indicate that host genetics and microbiota interact to shape muscle phenotype and meat quality outcomes.

More recently, evidence has extended the gut–muscle axis to include meat flavor. Modulation of the gut microbiota via probiotic supplementation increased the levels of flavor-associated nucleotides and umami-enhancing amino acids in muscle of finishing pigs, while reducing drip loss and thereby preserving flavor compounds [118]. This suggests that microbiota-mediated effects extend beyond structural traits to sensory properties, further emphasizing their relevance in meat production.

Despite these advances, several inconsistencies and knowledge gaps remain. One key issue is the dual role of microbiota in promoting both beneficial and detrimental outcomes. While certain microbes such as *L. reuteri* enhance meat quality, others such as *P. copri* induce inflammation and undesirable fat accumulation [111,113]. Moreover, increased intramuscular fat is generally associated with improved meat quality, but excessive fat deposition compromises carcass value, highlighting a trade-off that remains poorly understood. Similarly, the effects of microbiota on muscle fiber composition are not uniform, with some microbial communities promoting oxidative fibers and others glycolytic hypertrophy [101,114]. These discrepancies likely reflect differences in microbial metabolic outputs and host responses.

A unifying explanation for these divergent findings lies in the functional heterogeneity of microbial metabolites and their associated signaling pathways. SCFA such as valerate promote muscle growth and differentiation via GPR43-mediated activation of the Akt/mTOR signaling pathway [101]. In contrast, metabolites such as TMAO stimulate lipogenesis through transcriptional regulators [115], while endotoxins such as LPS trigger inflammatory pathways via TLR4 [113]. In addition, host genetic background and breed-specific traits influence both microbiota composition and muscle metabolism, further contributing to variability across studies [116,117]. Differences in experimental models, including species, housing conditions, and use of germ-free animals, may also affect the generalizability of findings. Taken together, these observations in pigs support a conceptual framework in which diet and host genetics shape gut microbial communities, which in turn produce metabolites that act on host signaling pathways to regulate muscle development, fiber composition, and lipid deposition. These processes ultimately determine meat quality traits, including tenderness, marbling, and flavor. Importantly, bidirectional interactions exist, as host physiology also influences microbial composition, creating a dynamic regulatory system.

Despite substantial progress, several important questions remain unresolved with regard to a functional gut–muscle axis in pigs. Amongst these, the identification of key microbial taxa and metabolites that consistently improve meat quality across breeds and production systems remains a major challenge. A further question concerns the precise molecular mechanisms linking microbial metabolites to host gene regulation, and this requires further elucidation. In addition, strategies are needed to optimize intramuscular fat deposition without promoting excessive overall adiposity. Moreover, the determinants of microbiota-driven shifts in muscle fiber type are still poorly understood. Finally, translating experimental findings into practical and stable microbiome-based interventions for pork production will require a deeper understanding of host–microbiome interactions and long-term system stability.

From a comparative perspective, several mechanisms identified in pigs appear to be conserved across livestock species. In particular, SCFA-mediated activation of Akt/mTOR signaling has also been reported in poultry and indirectly suggested in ruminants. However, pigs currently provide the strongest causal evidence for microbiota-mediated regulation of muscle development because both FMT and single-strain validation studies have demonstrated phenotype transfer. Another distinguishing feature of pigs is that microbial metabolites involved in gut–muscle communication are predominantly generated in the hindgut, whereas in ruminants similar metabolites originate mainly from ruminal fermentation. Therefore, caution is required when extrapolating mechanistic findings between monogastric and ruminant species.

### 3.2. Poultry

Like in pigs, the available evidence from studies in poultry, as summarized in Table 2, supports the existence of a functional gut–muscle axis. A key finding from these studies is that the gut microbiota can modulate skeletal muscle phenotype and growth. For instance, cecal microbiota transplantation from Jingyuan to Arbor Acres chickens resulted in the transfer of muscle characteristics, including increased fiber diameter and enhanced expression of genes related to oxidative fiber type, mitochondrial function, and glucose metabolism [119]. These effects were accompanied by an increased abundance of *L.* spp. (including *L. plantarum*, *L. salivarius*, and *L. ingluviei*) and elevated concentrations of SCFA in the cecum [119]. Importantly, administration of these *L.* strains individually or in combination reproduced similar phenotypic and molecular changes, providing strong evidence for a causal role of specific microbial taxa and their metabolites in regulating muscle traits via the gut–muscle axis [119].

A broader body of probiotic studies supports these findings and highlights converging mechanistic pathways. For example, post-hatch supplementation with *Enterococcus (E.) faecium* increased pectoralis muscle weight in broilers, an effect associated with enhanced capillarization, increased myofiber size, and altered expression of key myogenic regulators, including increased IGF1 and PAX7 and reduced myogenic factor 5 expression [120]. Given the role of PAX7 in the proliferation and maintenance of muscle progenitor cells [121], these results indicate that microbiota-modulating interventions can influence early myogenic programming. Similarly, supplementation with *Bacillus (B.) subtilis* induced a shift from type II to type I muscle fibers in broilers, likely mediated through activation of the AMPK/SIRT1/PGC-1α signaling pathway, as evidenced by increased expression of phosphorylated AMPK, SIRT1, and PGC-1α proteins [122]. This fiber-type transformation was associated with improved meat quality traits, including reduced drip loss, cooking loss, and shear force [122]. Additional studies demonstrate that modulation of the gut microbiota can also affect muscle metabolism and composition via altered lipid metabolism. For instance, supplementation with *C. butyricum* changed cecal microbiota composition and increased intramuscular fat content through enhanced lipogenic enzyme activity [123]. Similarly, feeding essential oils (thymol, carvacrol, and cinnamaldehyde) increased the abundance of SCFA-producing bacteria. This change was associated with activation of the IGF1/AKT/mTOR signaling pathway and the Nrf2 antioxidant system, leading to improved muscle fiber development and meat quality [124,125].

**Table 2:** Studies providing evidence for the regulation of muscle function and meat quality via the gut–muscle axis in poultry.

Breed, Sex	Experimental Approach	Slaughter Age	Main Effects (*p* < 0.05)	Ref.
Chickens (Chinese JY, AA), male)	- Cecal MT from JY to AA chickens: 10^8^ CFU/mL suspension of cecal content by gavage at a volume of 1.0 mL from the age of 22–42 d; control group: equal volume of sterilized PBS via gavage - Supplementation of *L.* spp. (*L. plantarum*, *L. ingluviei* and *L. salivarius*, 10^8^ CFU/mL, 1:1:1 mixed) via gavage (1 mL/d) from the age of 22–42 d; control group: equal volume of sterilized PBS via gavage	d 42 of age	- Cecal MT from JY to AA chickens caused a transfer of *pectoralis* muscle characteristics (increased fiber diameter) - Supplementation of *L.* spp.: ↑ expression of genes related to the oxidative fiber phenotype, ↑ fiber diameter and density	[119]
Chickens (Cobb 500, male)	Post-hatch application of *E. faecium* AL41: suspension with CFU of EF in 0.2 mL PBS was applied per os from d 1 to 7; control group: equal volume of PBS	d 5, 8, and 12 of age	↑ pectoralis muscle weights and myonuclei number per fiber, ↑ capillarization, ↑ myofiber size in *pectoralis* muscle	[120]
Chickens (AA, male)	Supplementation of *B. subtilis* (3.2 × 10^9^ CFU/g) via the diet (0, 300 and 500 mg/kg diet); control group: diet without *B. subtilis*	d 35 of age	↑ type II to type I muscle fiber transition, ↑ antioxidant capacity, improved muscular pH, meat color, water holding capacity and shear force in thigh muscle	[122]
Chickens (Ross 308, male)	Supplementation of *C. butyricum* (0 or 1 × 10^9^ cfu/kg diet) and *E. faecium* (0 or 2 × 10^9^ cfu/kg diet) via the diet; control group: diet without *C. butyricum* or *E. faecium*	d 42 of age	↑ intramuscular fat content and expression and activity of lipoprotein lipase in the breast muscle	[123]
Chickens (AA, male)	Supplementation of essential oils (blend of 200 mg cinnamaldehyde, 200 mg carvacrol and 100 mg thymol per kg diet) via the diet; control group: basal diet without essential oils	d 48 of age	- improved meat color, reduced shear force - ↑ breast muscle fiber development	[124]
Chickens (Ross 308, mixed sexes)	Supplementation of bile acids (250 mg/kg diet consisting of 73.2% HDCA, 19.8% CDCA and 3.9% HCA) via the diet; control group: basal diet without bile acids	d 42 of age	↑ breast muscle mass and myofiber diameter, ↑ RNA and DNA concentrations and expression of IGF-2 and FXR in the breast muscle	[126]
Ducks (Zhijiang, mixed sexes)	Supplementation of bile acids (250 mg/kg diet consisting of 73.2% HDCA, 19.8% CDCA and 3.9% HCA) together with a high fat-diet; control group: basal diet without bile acids	d 50 of age	↑ breast muscle weight and percentage, ↓ abdominal fat weight, ↑ cross-sectional area of breast muscle fibers	[127]
Ducks (Cherry Valley, unknown sex)	Supplementation of *L. plantarum* (20 g/kg diet) (2 × 10^10^ CFU/g) via the diet; control group: basal diet without *L. plantarum*	d 42 of age	↑ levels of pentanal, hexanal, heptanal, 1-octen-3-ol, 2,3-octanedione, and 2-pentylfuran	[128]

↑: Increase, ↓: Decrease. Abbreviations: AA, Arbor Acres; CDCA, chenodeoxycholic acid; HCA; hyocholic acid; HDCA, hyodeoxycholic acid; JY, Jingyuan.

Despite this general agreement on the importance of the gut–muscle axis, several inconsistencies remain. Not all studies directly assess changes in gut microbiota composition, limiting the ability to distinguish microbiota-mediated effects from direct effects of interventions. For example, although the *B. subtilis* study demonstrated clear changes in muscle signaling pathways and phenotype, it did not characterize the gut microbiota [122]. Furthermore, metabolic outcomes vary across studies as follows: some interventions promote oxidative metabolism and fiber-type switching [119,122], whereas others enhance lipogenesis and intramuscular fat deposition [123]. There is also no consistent microbial signature across studies; while *L.* spp. are frequently implicated [119], other studies report shifts in broader microbial communities or distinct taxa in response to different interventions, including bile acids or essential oils [124,125,126,127]. Finally, the degree of mechanistic insight varies, with some studies providing detailed molecular evidence and others relying primarily on associative observations.

These discrepancies likely arise from several factors. Host-related variables, including species (chickens vs. ducks), breed, and developmental stage, likely influence the response to microbiota modulation. For example, beneficial effects of bile acids on muscle growth have been reported in both broilers [126] and ducks [127], but these effects are accompanied by species-specific changes in microbiota composition, suggesting context-dependent responses. In addition, differences in intervention type (e.g., microbiota transplantation, single-strain probiotics, multi-strain formulations, or dietary additives), dosage, and timing contribute to variability. The inherent complexity of the gut microbiota further complicates interpretation, as functional redundancy allows different microbial communities to produce similar metabolites, particularly SCFA. Thus, microbial metabolic output rather than taxonomic composition per se may be the primary determinant of host responses. Moreover, host metabolic context, such as diet composition, may influence whether microbiota-mediated effects favor oxidative metabolism or lipid accumulation in muscle.

On the basis of the available evidence, a conceptual framework for the gut–muscle axis in poultry can be proposed. Microbiota-modulating interventions alter the composition and functional capacity of the gut microbiota, leading to changes in the production of metabolites such as SCFA and bile acids [119,124,125,126,127]. These metabolites enter the systemic circulation and act as signaling molecules by interacting with host receptors, including G protein-coupled receptors and nuclear receptors such as FXR. For example, bile acid supplementation to broilers increased muscle expression of FXR and IGF2, suggesting activation of an FXR-mediated IGF2 pathway that promotes muscle growth [126]. These signals converge on key intracellular pathways in muscle tissue, including IGF1/IGF2-dependent growth signaling, AMPK/SIRT1/PGC-1α-mediated regulation of mitochondrial function and fiber-type switching, mTOR signaling for protein synthesis, and Nrf2 pathways for antioxidant defense [120,122,125,126]. The integration of these pathways ultimately determines muscle fiber characteristics, lipid metabolism, and meat quality traits. In addition, modulation of microbial metabolism influences the production of volatile flavor compounds, such as pentanal, hexanal, heptanal, 1-octen-3-ol, 2,3-octanedione, and 2-pentylfuran, and metabolic intermediates that contribute to meat flavor, as demonstrated in ducks supplemented with lactic acid bacteria [128].

While progress has been achieved, several major questions in poultry have yet to be resolved. The specific metabolites mediating communication along the gut–muscle axis have not been conclusively identified, and it remains unclear whether SCFA alone are sufficient or whether synergistic interactions among multiple microbial metabolites are required. It is also unclear whether specific microbial taxa are essential or whether functional redundancy allows different microbial communities to produce similar effects. Furthermore, the relative contribution of direct versus microbiota-mediated effects of dietary components, such as bile acids and essential oils, remains to be determined. Another unresolved issue is the divergence in metabolic outcomes, particularly the balance between oxidative metabolism and lipid deposition in muscle. Finally, the lack of integrative multi-omics approaches limits the ability to establish causal relationships and to develop predictive models of the gut–muscle axis.

Comparison of poultry and pig studies reveals substantial convergence in the signaling pathways involved in gut–muscle communication. In both species, microbiota-derived metabolites are linked to activation of Akt-/mTOR-associated anabolic signaling and modulation of muscle fiber type. However, poultry studies more frequently report activation of AMPK/SIRT1/PGC-1α signaling and promotion of oxidative muscle metabolism. Similar to pigs, most microbial fermentation occurs in the hindgut, supporting a central role for cecal SCFA production. Together, the findings from monogastric livestock suggest that SCFA-mediated regulation of muscle growth and fiber characteristics represents a conserved component of the gut–muscle axis.

### 3.3. Ruminants

Based on studies in ruminant livestock as summarized in Table 3, the present body of evidence supports the existence of a functional gut–muscle axis in ruminants, whereby gut microbial communities and their metabolites contribute to the regulation of muscle development and meat quality. Collectively, the reviewed studies indicate that this axis operates through complex interactions between microbial composition, metabolite production, and host gene expression.

A recurring finding across studies is the association between specific gut microbial taxa and muscle-related gene expression. For example, comparative analyses in cattle revealed that rectal microbial genera such as *Bacteroides uniformis*, *Roseburia inulinivorans*, *Bacteroides vulgatus*, *C. catus*, *Eubacterium rectale*, and *Faecalibacterium prausnitzii* are positively correlated with genes involved in muscle growth, fiber composition, and lipid metabolism, including myostatin, myosin light chains, troponins, and fatty acid binding proteins [129]. These associations, together with correlations between microbial metabolites such as isobutyrate, isovalerate, valerate, and caproate and the same bacterial taxa, suggest that microbial metabolic outputs may act as signaling molecules influencing muscle physiology. Similarly, associations between gut metabolites and muscle gene expression in Liangshan black sheep and Meigu goats reported by Chen et al. [130], including links between adenylate cyclase 1 and solute carrier family 38 member 4 with metabolites such as L-tyrosine ethyl ester and pelargonidin 3-O-glucoside, further support the role of microbially modulated metabolites in shaping muscle phenotype and meat quality traits. These findings are consistent with known roles of these genes in muscle differentiation and amino acid transport [131,132].

In addition to SCFA and amino acid metabolites, bile acids have emerged as important mediators of the gut–muscle axis. Breed-specific differences in bile acid metabolism, as demonstrated in Dorper and Tan sheep [133,134,135], were associated with variation in both muscle fiber characteristics and carcass traits. For instance, higher concentrations of the secondary bile acid DCA in Tan sheep were positively correlated with muscle fiber density, whereas primary bile acids such as glycocholic acid (GCA) in Dorper sheep were associated with carcass weight and fat deposition [135]. Experimental evidence further supports these observations, as supplementation with UDCA improved intramuscular fat deposition and carcass traits in Japanese Black cattle [136], while bile acid supplementation in lambs altered fat distribution and adipose gene expression [137]. Together, these findings suggest that microbiota-driven bile acid metabolism contributes to the regulation of muscle development and lipid deposition.

Another important aspect highlighted by the literature is the bidirectional nature of host–microbiome interactions. Evidence from myostatin gene-edited cattle indicates that host genetic modifications can reshape gut microbial composition and function, with differences observed in both cecal and colonic microbial communities [138]. The altered microbiota was enriched in pathways related to carbohydrate metabolism, while muscle transcriptomic analysis revealed an upregulation of genes involved in glycolysis, suggesting enhanced anaerobic glucose metabolism [138]. These findings imply that host genetics not only determine muscle phenotype directly but may also influence muscle metabolism indirectly through microbiota-mediated mechanisms.

Dietary interventions, particularly probiotic supplementation, provide further support for a functional gut–muscle axis and offer practical avenues for its manipulation in ruminant livestock. Supplementation with *C. butyricum* has been shown to improve growth performance, muscle mass, and meat quality traits in lambs, accompanied by activation of the IGF1/Akt/mTOR signaling pathway and reduced expression of protein degradation genes [139]. Similarly, supplementation with *L. casei* improved muscle development, reduced fat deposition, and enhanced meat quality traits such as tenderness and water-holding capacity in lambs [140]. Feeding a probiotic mixture containing *L. plantarum* and *L. casei* increased concentrations of SCFA (propionate, butyrate, and valerate), promoted a shift toward oxidative (type I) muscle fibers, and improved meat quality parameters in lambs [141]. These findings are consistent with mechanistic evidence showing that butyrate can regulate muscle fiber gene expression, promoting oxidative fiber types in mice [142]. Additional studies confirmed that probiotic supplementation modulates key signaling pathways, including MAPK and FOXO, and alters gene expression related to muscle development and fat deposition in lambs [143].

**Table 3:** Studies providing evidence for the regulation of muscle function and meat quality via the gut–muscle axis in ruminant livestock.

Breed, Sex	Experimental Approach	Slaughter Age	Main Effects (*p* < 0.05)	Ref.
Cattle (Angus, Chinese Simmental, male)	Comparative correlation analysis between metagenomic and transcriptomic data between two cattle breeds fed with the same diet for at least 10 mo	18 mo of age	Abundance of 17 bacterial species in feces was positively correlated to muscle and fat metabolism genes	[129]
Goat (Meigu, male) Sheep (Liangshan black, male)	Correlation analysis between metabolomic and muscle transcriptomic data in Meigu goats and Liangshan black sheep fed with the same feed	Approx. 24 mo of age	17 genes in muscle and 19 gut metabolites were significantly correlated with more than eight meat quality parameters across multiple gut sites	[130]
Sheep (Tan, Dorper, mixed sexes)	Correlation analysis between metagenomic (rumen, hindgut) and metabolomic data in two sheep breeds fed with the same feed	Approx. 8 mo of age	- Nicotinic acid was negatively correlated with body weight - 2-deoxyadenosine was positively correlated with fatty acids content - Negative correlation between Phascolarctobacterium and deoxycytidine levels in the hindgut - Deoxycytidine was positively correlated with body weight, protein, and amino acid content	[133]
Sheep (Tan, Dorper, unknown sex)	Correlation analysis between metagenomic (rumen, duodenum, and colon) and meat quality data in two different sheep breeds fed with the same feed	8 mo of age	- Abundance of *Achromobacter xylosoxidans*, *Mageeibacillus indolicus*, and *Mycobacterium dioxanotrophicus* were positively correlated with C12:0 - Abundance of *Methanobrevibacter millerae*, *Bacteroidales bacterium CF*, and *Bacteroides coprosuis* were negatively correlated with C12:0	[134]
Sheep (Tan, Dorper, mixed sexes)	Correlation analysis between metagenomic and muscle transcriptomic data in two sheep breeds fed with the same feed	8 mo of age	DHCA was strongly correlated with *g_Ruminococcaceae_UCG-014*, *ENSOARG00000001393*, and *ENSOARG00000016726*, muscle fiber density and diameter - GCA was associated with *g_Lachnoclostridium_10*, *g_Rikenellaceae_RC9_gut_group*, *ENSOARG0000001232*, carcass weight, and net meat weight	[135]
Cattle (Wagyu, heifers)	Supplementation of UDCA (2.5 g/d) via the diet 24 times during 7 mo; control group: basal diet without UDCA	29 mo of age	UDCA: ↑ meat quality grade and marbling, ↑ percentage of ether extract in the lean mass, ↑ L* (lightness) value of muscles	[136]
Sheep (Tan lambs, unknown sex)	Supplementation of either HCA or ruminally protected HDCA at 0.04% of body weight; control group: basal diet without HCA or HDCA	Not provided	- HCA: ↓ body fat - HDCA: ↓ tail fat weight - HCA and HDCA: ↓ tail fat ratio	[137]
Cattle (MSTN gene-edited and wildtype cattle, Mongolian breed, male)	Influence of MSTN deletion; control group: wildtype cattle	At approx. 24 mo of age	Increased abundance of Bacteroides and carbohydrate-active enzymes was linked to enhanced glycolysis/gluconeogenesis and carbohydrate metabolism	[138]
Sheep (Dorper (♂) × Small Tailed Han sheep (♀) crossed ewe lambs, unknown sex)	Supplementation of *C. butyricum* (2.5 × 10^8^ cfu/g, 5 g/d) via the diet for 90 d; control group: basal diet without *C. butyricum*	180 d of age	↑ growth performance, muscle mass, and muscle fiber diameter; ↓ shear force value of meat; ↑ protein synthesis	[139]
Sheep (Sunit lambs, male)	Supplementation of 6, 12 and 24 g/d *L.* spp. mixture (*L. casei HM-09* and *L. plantarum HM-10*; 1.5 × 10^9^ CFU/g) via the diet for 90 d; control group: basal diet without *L.* spp. mixture	180 d of age	↑ loin muscle area, ↓ tail and visceral fat deposition; ↓ shear force, cooking loss and intramuscular fat content in the *longissimus thoracis* and *biceps femoris* muscles	[140]
Sheep (Sunit lambs, mixed sexes)	Supplementation of 2 g/d *L.* spp. mixture (*L. casei HM-09* and *L. plantarum HM-10*; 1.5 × 10^9^ CFU/g) via the diet for 90 d; control group: basal diet without *L.* spp. mixture	180 d of age	↑ number and area ratio of type I muscle fibers; ↑ mRNA levels of MyHC IIA and the activity of malate dehydrogenase and succinate dehydrogenase; ↓ cooking loss, pH_24h_, and shear force	[141]
Sheep (Sunit lambs, mixed sexes)	Supplementation of 10 g probiotic mixture/kg concentrate offered at 1.5% of live weight; the probiotic mixture consisted of a 1:1 mixture of *L. casei Zhang* and *L. plantarum P-8* (1.5 × 10^9^ CFU/g); control group: equal amount of concentrate without probiotic mixture	180 d of age	↑ area, pH_24h_ and intramuscular fat content of *longissimus thoracis* muscle; ↓ cooking loss and meat shear force of *longissimus thoracis* muscle	[143]
Goats (crossbred Thai native ♀ × Anglo-Nubian ♂, male)	Supplementation of 2.5 or 5 g/d of *L. acidophilus* (2.0 × 10^12^ CFU/g) and *S. cerevisiae* (5.0 × 10^11^ CFU/g) via the diet; control group: basal diet without *L.* spp. mixture	Not provided	↑ a* value of *longissimus thoracis et lumborum* Supplementation of 5 g/d of probiotic mixture: ↑ nutritive value index [=(C18:0 + C18:1)/C16:0)] of muscle	[144]

↑: Increase, ↓: Decrease. Abbreviations: HCA; hyocholic acid; HDCA, hyodeoxycholic acid; MSTN, myostatin; UDCA, ursodeoxycholic acid.

Beyond effects on muscle structure and growth, probiotic supplementation also influences muscle lipid metabolism in ruminants. In goats, supplementation with *L. acidophilus* and *Saccharomyces cerevisiae* increased intramuscular fat content and concentrations of conjugated linoleic acids (CLA) in muscle [144]. This effect is likely mediated by enhanced rumen biohydrogenation, a microbial process driven by bacteria such as *Butyrivibrio fibrisolvens*, *Ruminococcus albus*, *Ruminococcus flavefaciens*, and *Fibrobacter succinogenes* [145,146,147,148]. These findings highlight the importance of rumen microbial activity in shaping fatty acid composition and meat quality.

Despite these consistent patterns, several uncertainties and inconsistencies remain. A key limitation of the current literature in ruminant livestock is the reliance on associative data, which precludes definitive conclusions regarding causality. The predominance of correlative studies partly reflects the inherent complexity of the rumen ecosystem and the technical challenges associated with causal microbiome manipulation in ruminants. Moreover, many studies focus on hindgut microbiota (e.g., rectum or colon), despite the rumen being the primary site of microbial fermentation in ruminants. As acknowledged in previous work [129], the relative contribution of rumen versus hindgut microbiota to muscle regulation remains unclear. A key unresolved issue concerns the relative roles of ruminal and hindgut microbiota in regulating the gut–muscle axis in ruminants. Rumen is the primary site of microbial fermentation and accounts for the majority of SCFA production, microbial protein synthesis, and lipid biohydrogenation. Consequently, ruminal microbial communities are likely to exert substantial effects on host energy supply, amino acid availability, and fatty acid metabolism, all of which are closely linked to muscle growth and carcass composition. In contrast, the hindgut contributes a smaller proportion of total fermentation but may generate distinct metabolites, including secondary bile acids, aromatic amino acid metabolites, and other microbial signaling molecules capable of entering the systemic circulation. Thus, ruminal microbiota may primarily influence muscle development through effects on nutrient harvesting and metabolic substrate supply, whereas hindgut microbiota may play a more prominent signaling role through the production of regulatory metabolites that directly affect host gene expression and cellular signaling pathways. Rather than acting independently, both microbial compartments likely function as interconnected components of a unified gut–muscle regulatory network. Future studies should therefore simultaneously characterize ruminal and hindgut microbial communities, metabolite profiles, and muscle phenotypes in order to determine their relative and potentially complementary contributions to muscle development and meat quality.

Furthermore, breed-specific differences, variation in dietary conditions, and methodological heterogeneity across studies complicate direct comparisons and may explain discrepancies in reported outcomes. A further challenge in establishing causality in ruminants relates to the unique complexity of the rumen ecosystem. Unlike monogastric species, the rumen harbors a dense and highly diverse consortium of bacteria, archaea, protozoa, fungi, and bacteriophages that interact through intricate cross-feeding networks and collectively drive fermentation processes. Consequently, manipulation of individual microbial taxa often results in secondary changes throughout the microbial community and its metabolic outputs, making it difficult to attribute host responses to specific microorganisms or metabolites. In addition, causal approaches commonly used in laboratory rodents, such as FMT, are technically more challenging in ruminants. Successful rumen microbiota transplantation requires the transfer and stable establishment of a complex anaerobic microbial community within an already mature and resilient rumen ecosystem, and engraftment efficiency can vary considerably among recipients. Likewise, the use of germ-free or gnotobiotic ruminant models is associated with substantial logistical, technical, and financial constraints, greatly limiting experimental opportunities. These factors help explain why most available studies in ruminants rely on multi-omics association analyses, dietary interventions, or probiotic supplementation rather than direct causal verification through microbiota transplantation or single-strain colonization experiments.

To facilitate interpretation of the heterogeneous literature, a unified analytical framework can be proposed in which variation in gut–muscle axis outcomes is viewed as the consequence of interacting host, environmental, and methodological factors. Among these, diet is likely the dominant determinant because dietary composition directly alters microbial substrate availability and consequently the production of SCFA, bile acids, amino acid metabolites, and lipid-derived signaling molecules. Host genetics and breed further influence both microbiota composition and host responsiveness to microbial metabolites, thereby contributing to differences in muscle fiber characteristics, growth rate, and fat deposition. Animal age and developmental stage may additionally modify microbiota maturation, metabolic requirements, and muscle plasticity, resulting in age-dependent responses to microbial interventions. Variation in probiotic dosage, supplementation duration, or metabolite exposure can influence both the magnitude and direction of physiological responses. Furthermore, the anatomical location of microbiota sampling represents a major source of inconsistency, particularly in ruminants, because ruminal, cecal, colonic, and fecal microbial communities differ substantially in taxonomic composition and metabolic activity. These factors ultimately converge by altering microbial metabolite production and host signaling pathways, thereby influencing muscle growth, fiber-type composition, lipid deposition, and meat quality traits. Adoption of this framework may improve comparability across studies and facilitate the identification of biological mechanisms that are conserved across different experimental settings.

These considerations suggest that the gut–muscle axis in ruminants should be conceptualized as an integrated, multi-level system. Importantly, this system should be viewed as comprising at least two interacting microbial compartments, namely the rumen and hindgut, each contributing distinct but potentially complementary metabolite pools to host–muscle communication. In such a framework, diet and host genetics shape microbial community composition and function across different gut compartments, leading to the production of metabolites such as SCFA, bile acids, and lipid intermediates. These metabolites influence host tissues via signaling pathways including IGF1/Akt/mTOR, MAPK, and FOXO, ultimately regulating muscle growth, fiber type composition, and fat deposition. At the same time, host genetic factors, as illustrated by myostatin-related effects [138], feed back on the microbiota, creating a dynamic regulatory loop.

Several key questions regarding the existence of a functional gut–muscle axis in ruminant livestock remain to be addressed. First, the specific contribution of rumen microbiota to muscle gene expression and meat quality requires further investigation. Second, mechanistic studies are needed to establish causal relationships between microbial taxa, metabolites, and muscle phenotypes. Third, the interaction between host genetics and microbiota, particularly in the context of breed-specific traits, remains insufficiently understood. Finally, more standardized experimental approaches and integrated multi-omics analyses will be necessary to advance from descriptive associations to predictive and mechanistic models of the gut–muscle axis.

In contrast to pigs and poultry, evidence in ruminants is derived largely from association studies rather than direct microbiome transfer experiments. Nevertheless, several regulatory mechanisms appear conserved across species. SCFA and bile acids are consistently linked to muscle-related pathways, including Akt/mTOR, MAPK, and mitochondrial metabolism. A major physiological difference is that SCFA production in monogastrics occurs primarily in the hindgut, whereas in ruminants fermentation and SCFA production occur predominantly in the rumen. Consequently, the anatomical site of microbial metabolite production and absorption differs substantially between production systems. Similarly, host–genotype–microbiota interactions appear to be conserved, although their manifestation may differ. For example, myostatin deficiency alters microbial composition in both pigs and cattle, yet the affected microbial taxa and metabolic pathways are not identical, suggesting species-specific host–microbiome adaptations.

### 3.4. Cross-Species Comparison of the Gut–Muscle Axis

Despite species-specific differences, several conserved principles emerge across livestock species, as summarized in Table 4. First, microbial metabolites rather than taxonomic composition appear to be the primary drivers of gut–muscle communication. Second, SCFA and bile acids consistently emerge as central mediators linking microbial activity to muscle growth, muscle fiber composition, and meat quality. Third, downstream activation of anabolic signaling pathways, particularly Akt/mTOR, represents a recurring mechanism across pigs, poultry, and ruminants. At the same time, important species-specific differences exist. In monogastric animals, microbial fermentation occurs primarily in the hindgut, whereas in ruminants it occurs mainly in the rumen. Consequently, microbial metabolite production, absorption kinetics, and host exposure differ substantially between production systems. Moreover, causal evidence derived from FMT and targeted probiotic interventions is currently available mainly in pigs and poultry, whereas evidence in ruminants remains predominantly associative. Host genetics further contribute to interspecies variation. For example, myostatin deficiency alters gut microbial communities in both pigs and cattle, but the specific microbial responses and metabolic consequences differ between species. Collectively, these observations support a model in which conserved metabolite-dependent signaling pathways operate across livestock species, whereas microbial taxa, fermentation sites, and host genetic interactions exhibit substantial species-specific variation.

## 4. Conclusions and Future Perspectives

The evidence presented across pigs, poultry, and ruminant livestock provides strong support for the existence of a functional gut–muscle axis that contributes to the regulation of muscle growth, fiber composition, intramuscular fat deposition, and ultimately meat quality traits. A central conclusion emerging from this body of work is that the gut microbiota acts as a metabolically active interface between diet, host genetics, and muscle physiology. Through the production of bioactive metabolites such as SCFA, bile acids, TMAO, and lipid intermediates, gut microbial communities influence key host signaling pathways, including IGF/Akt/mTOR, AMPK/SIRT1/PGC-1α, MAPK, FOXO, and inflammatory cascades. These pathways converge to regulate myogenesis, muscle fiber type specification, mitochondrial function, and lipid metabolism, thereby determining economically important production traits such as growth efficiency, carcass composition, tenderness, marbling, and flavor. A major conceptual advance is the recognition that microbial function—particularly metabolite production—rather than taxonomic composition per se is the primary driver of gut–muscle communication. Across species, different microbial communities often lead to similar phenotypic outcomes, suggesting a high degree of functional redundancy within the microbiome. At the same time, the effects of the gut microbiota are not universally beneficial. Certain taxa and metabolites promote desirable traits such as improved marbling and tenderness, whereas others induce inflammation, excessive fat deposition, or reduced lean meat yield. This highlights a critical trade-off between optimizing meat quality and maintaining production efficiency, which remains insufficiently understood. Another key conclusion is that the gut–muscle axis operates as a dynamic, bidirectional system. While diet and host genetics shape microbial composition and activity, host metabolic status and genetic background feed back to influence the microbiota. This interaction is particularly evident in breed-specific differences and in genetically modified animals, underscoring the importance of considering host–microbiome interactions as an integrated system rather than independent factors. Despite these advances, the level of mechanistic resolution varies considerably among livestock species. While causal relationships have been demonstrated in pigs and poultry through microbiota transplantation and targeted interventions, evidence in ruminants is still largely associative and requires further validation.

Looking ahead, several key priorities should guide future research in this field. Future studies should prioritize microbial metabolites for which preliminary mechanistic evidence already exists. Among these, valerate, IPA, and urolithins are particularly promising candidates. We hypothesize that valerate primarily regulates muscle fiber hypertrophy through GPR43-Akt/mTOR signaling, whereas IPA and urolithins mainly enhance muscle growth by suppressing inflammatory and proteolytic pathways. These hypotheses should be tested across livestock species using targeted metabolite supplementation and microbiota manipulation approaches. To establish causality, future studies should employ a tiered experimental framework. Candidate metabolites should first be evaluated in primary livestock myoblast cultures or muscle organoid systems. Subsequently, findings should be validated in vivo using FMT, defined microbial consortia, probiotic interventions, or targeted metabolite supplementation. Mechanistic confirmation should be obtained through receptor inhibition or gene-editing approaches targeting pathways such as GPR43, FXR, TGR5, Akt/mTOR, and FOXO. Standardized multi-omics pipelines should integrate shotgun metagenomics to identify metabolite-producing microorganisms, targeted metabolomics of intestinal content, plasma and muscle tissue, muscle transcriptomics and proteomics, and detailed phenotyping of muscle fiber composition, intramuscular fat content and meat quality traits. Integration of these datasets may enable the identification of predictive microbe–metabolite–host interaction networks. In pigs and poultry, future work should focus on identifying microbial consortia and metabolites capable of reproducibly improving marbling, tenderness, and carcass quality under commercial production conditions. In contrast, research in ruminants should prioritize the development of causal experimental models, including rumen microbiota transplantation and early-life microbiome programming approaches, in order to move beyond the currently predominant association-based evidence.

Despite the considerable progress in elucidating the gut–muscle axis, several challenges currently limit the translation of microbiome-based strategies into commercial livestock production systems. One major bottleneck is the stability and persistence of microbiome interventions under field conditions. Probiotic strains that produce beneficial effects under controlled experimental settings may fail to establish long-term colonization in commercial herds and flocks due to differences in diet composition, housing conditions, health status, environmental stressors, and competition with resident microbial communities. Moreover, the efficacy of microbial interventions is often host-specific, with considerable variation observed between breeds, genetic lines, developmental stages, and production systems. Consequently, microbial products that improve muscle growth or meat quality in one population may exhibit reduced or inconsistent efficacy in another. Economic and regulatory considerations represent additional barriers to implementation. The large-scale production, stabilization, storage, and delivery of live microbial products must be economically feasible for producers while maintaining microbial viability throughout the supply chain. Furthermore, regulatory approval of novel microbial feed additives often requires extensive demonstration of safety, product consistency, and efficacy, which may increase development costs and delay commercialization. Long-term studies under commercial production conditions are therefore needed to evaluate both the biological effectiveness and economic viability of microbiome-targeted interventions. An additional challenge concerns the optimization of carcass composition. While many microbiome-based interventions increase intramuscular fat deposition and thereby improve marbling, tenderness, and flavor, excessive stimulation of adipogenesis may simultaneously reduce carcass lean percentage and production efficiency. Future research should therefore focus on identifying microbial taxa, microbial consortia, or metabolite profiles that selectively regulate muscle lipid metabolism without promoting excessive fat deposition in subcutaneous or visceral adipose tissues. One promising strategy involves screening combinations of probiotic strains with complementary metabolic functions, for example, strains that stimulate intramuscular adipocyte differentiation and marbling while simultaneously promoting muscle protein accretion, fatty acid oxidation, or lean tissue growth. Such targeted microbial consortia could potentially improve eating quality while minimizing adverse effects on carcass composition.

Another emerging research priority is the integration of host genetics and microbiome-based approaches. Accumulating evidence from pigs, poultry, and ruminants indicates that host genotype can influence microbial community composition and metabolic activity, whereas microbiota-derived metabolites regulate signaling pathways involved in muscle growth, fiber-type specification, and lipid deposition. These observations suggest that genetic selection and microbiome management should not be viewed as separate strategies but rather as complementary components of precision livestock production. In the future, breeding programs may incorporate microbiome-associated traits alongside conventional production traits such as growth rate, feed efficiency, carcass yield, and meat quality. The identification of host genetic variants linked to favorable microbial communities or metabolite profiles could facilitate the selection of animals with an enhanced capacity to establish microbiota that promote desirable muscle phenotypes. Conversely, microbiome-targeted nutritional interventions could be tailored to specific genetic backgrounds, thereby maximizing host responsiveness to microbial modulation. Particularly promising is the concept of genotype-specific microbiome engineering, whereby microbial interventions are designed according to the genetic predisposition of individual breeds or production lines. For example, animals genetically predisposed to excessive fat deposition may benefit from microbial consortia that favor muscle protein synthesis and oxidative metabolism, whereas lean genotypes with limited marbling potential could be targeted with microbiota that promote intramuscular fat deposition without increasing overall adiposity. Such integrated host genetics–microbiome approaches may ultimately enable the simultaneous optimization of growth performance, carcass lean rate, feed efficiency, and eating quality.

From an applied perspective, translating current knowledge into practical strategies will be a key challenge. The development of precision microbiome interventions, such as tailored probiotics, synbiotics, postbiotics, microbial consortia, or dietary formulations, holds significant promise but requires a deeper understanding of microbiome stability, host specificity, production costs, regulatory requirements, and long-term efficacy under commercial conditions. Equally important will be the integration of host genetic selection with microbiome-based approaches. Combining genomic breeding strategies with targeted manipulation of microbial communities and metabolite production may provide synergistic opportunities to optimize muscle growth, carcass composition, meat quality, and feed efficiency. In particular, the development of microbial interventions that selectively enhance intramuscular fat deposition while preserving carcass leanness represents a promising objective for future precision livestock production systems.

In conclusion, the gut–muscle axis represents a fundamental regulatory system in livestock biology with significant implications for animal performance and meat quality. While substantial progress has been made, advancing from descriptive and associative findings to predictive, mechanism-based models will be essential to fully exploit the potential of microbiome-driven strategies in livestock production.

## Figures and Tables

**Table 1:** Studies providing evidence for the regulation of muscle function and meat quality via the gut–muscle axis in pigs.

Breed, Sex	Experimental Approach	Slaughter Age	Main Effects (*p* < 0.05)	Ref.
Obese J, lean L, mixed sexes	- FMT from J pigs and L pigs to antibiotics-treated mice	Pigs: 8 mo of age	- Mice receiving FMT from JP: ↑ lipid and triglyceride levels in muscle	[110]
Obese NX, lean DLY, male	- FMT from obese NX pigs to lean DLY pigs; 60 mL fresh fecal samples were mixed with 240 mL saline; transplantation every d for 14 wk - Supplementation of *L. reuteri* XL0930 (1 × 10^11^ CFU) via gavage every d for 60 d; control: equal volume of saline via gavae	150 d of age	- FMT from obese NX pigs to DLY pigs: ↑ muscle FA content, improved meat quality - *L. reuteri* XL0930 administration by gavage to lean DLY pigs: ↑ muscle FA content	[111]
Duroc, unknown sex	- Correlation analysis between gut microbiome and lean meat percentage - Supplementation of *P. copri* via gavage (100 μL of suspension (1 × 10^7^ CFU/μL) 3×/wk for 4 wk to germ free mice; control: chow diet without gavage	160 d of age	- *P. copri* abundance in the gut was positively associated with fat accumulation of pigs - *P. copri* isolated from experimental pigs administered to germ-free mice: ↑ host fat accumulation	[113]
Obese R, lean Y, unknown sex	FMT from obese R pigs and lean Y pigs to germ-free mice; mice were colonized with 0.05 mL of the porcine fecal suspension using a nasogastric tube, and 2 mL aliquot suspensions were spread on the fur of each foster mouse	20 wk of age	- FMT from either R pigs or Y pigs into germ-free mice: ↑ body fat mass, ↑ slow-contracting fiber proportion, ↓ fiber size and fast IIb fiber percentage in the muscle	[114]
Unknown breed and sex	Supplementation of TMAO (0 or 1 g/kg diet) for 149 d; control group: identical basal diet without TMAO	Approx. 210 d of age	↑ backfat thickness, ↑ intramuscular fat content	[115]
Chinese SB and LWL, male	Comparative analysis of muscle transcriptome and plasma metabolome of different pig breeds fed the same diet	210 ± 15 d of age	- Transcriptomics-based protein–protein interaction network analysis identified 25 key genes being associated with muscle development, fat deposition and meat quality in SB pigs - Metabolomic analysis identified several metabolites, primarily involved in fructose and mannose metabolism, amino acid biosynthesis, nucleotide sugar metabolism, and glucagon signaling pathways in SB pigs	[116]
Chinese SB and LWL, male	Comparative integrative analysis of multi-omics data of different pig breeds fed the same diet	210 ± 15 d of age	- In SB pigs: Peptostreptococcaceae and Rickettsiales were correlated with plasma PC(22:4), NAE(20:4), and LysoPC(24:1) and mRNA levels of EIF4E, MSTN, PPARGC1A, NR4A3, and SOCS1 - In LWL pigs: Corynebacterium and Streptococcaceae were correlated with transcript level of PPP1R3B and plasma 2-methyl-3-hydroxybutyric acid and 5-keto-gluconic acid	[117]
DLY, male	Supplementation of a probiotic mixture (1 g/kg diet) of *L. acidophilus* (1 × 10^6^ CFU/g) and *B. subtilis* (1 × 10^6^ CFU/g) via the diet for 49 d; control group: fed the same basal diet without probiotic mixture	Approx. 220 d	↑ flavor-associated nucleotides and umami-enhancing amino acids in *longissimus dorsi* muscle	[118]

↑: Increase, ↓: Decrease. Abbreviations: DLY, Duroc × Landrace × Yorkshire; J, Jinhua; L, landrace pigs; LWL, Large White × Landrace; NX, Ningxiang; R, Rongchang; SB, Songliao Black; TMAO, TMA-N-oxide; Y, Yorkshire.

**Table 4:** Cross-species comparison of the gut–muscle axis.

Species Group	Main Evidence Type	Key Microbial/Metabolic Mediators	Conserved Signaling Pathways	Main Outcomes	Level of Evidence
Pigs	FMT, probiotics, metabolite supplementation	SCFA (valerate), TMAO, *L. reuteri*	GPR43-Akt/mTOR, TLR4, mTOR	Intramuscular fat, fiber composition, marbling, flavor	Strong causal
Poultry	Cecal microbiota transplantation, probiotics	SCFA, bile acids, *L.* spp.	IGF/Akt/mTOR, AMPK/SIRT1/PGC-1α, FXR	Muscle growth, oxidative fibers, meat quality	Strong causal/moderate mechanistic
Ruminants	Multi-omics studies, probiotics	SCFA, bile acids, CLA intermediates	IGF/Akt/mTOR, MAPK, FOXO	Muscle growth, fat deposition, tenderness	Mainly associative
Across species	Multiple approaches	SCFA, bile acids	mTOR-related pathways, mitochondrial regulation	Muscle development and meat quality	Conserved evidence

Abbreviations: AMPK, AMP-activated protein kinase; CLA, conjugated linoleic acid; FMT, fecal microbiota transplantation; FOXO, forkhead box O; GPR, G-protein-coupled receptor; IGF, insulin-like growth factor; *L.*, *Lactobacillus*; MAPK, mitogen-activated protein kinase; mTOR, mammalian target of rapamycin; PGC-1α, peroxisome proliferator-activated receptor gamma coactivator 1-α; SCFA, short-chain fatty acids; SIRT, sirtuin; TLR, Toll-like receptor; TMAO, trimethylamine-N-oxide.

## Data Availability

No new data were created or analyzed in this study. Data sharing is not applicable.
